# Performance, acceptability, and impact of ambient listening scribe technology in an outpatient context: a mixed methods trial evaluation

**DOI:** 10.1186/s12913-025-13954-5

**Published:** 2026-01-08

**Authors:** Salim Memon, Adam Brand, Bianca Taylor, Adelaide Michael, Rachael Smithson

**Affiliations:** 1 General Medicine, Gold Coast Hospital and Health Service, Gold Coast, Australia; 2 Emergency Department, Gold Coast Hospital and Health Service, Gold Coast, Australia; 3 Digital Transformation and Research, Gold Coast Hospital and Health Service, Gold Coast, Australia; 4 Health Systems Research Group, Gold Coast Hospital and Health Service, Gold Coast, Australia

**Keywords:** Artificial intelligence, Ambient listening, Medical transcription, Scribe technology, Machine learning, Digital innovation

## Abstract

**Background:**

In 2024, Gold Coast Hospital and Health Service outpatient division initiated a 16-week trial of artificial intelligence (AI)-enabled ambient listening scribe technology. The objective of this pilot study was to evaluate scribe technology performance (according to dimensions of quality, utility and reliability) and its impact on the experience of outpatient clinicians and patients receiving care.

**Methods:**

A mixed method research design combined analysis of data from patient and staff surveys, staff interviews, scribe outputs and electronic medical records across a breadth of outpatient specialties.

**Results:**

By and large, ambient listening technology was associated with positive patient and staff experience. On average, 58% of scribe outputs were accepted without modification into the electronic outpatient note. There was limited evidence of bias in outputs, however there was some evidence of hallucination or incorrect outputs.

**Conclusions:**

Qualitative and quantitative data were internally consistent and demonstrated that ambient listening technology can (1) produce an accurate summary of outpatient appointments, (2) enhance clinical note quality and (3) improve both clinician and patient experience.

**Clinical trial number:**

Not applicable.

**Supplementary Information:**

The online version contains supplementary material available at 10.1186/s12913-025-13954-5.

## Abstract (significance)

What is already known on this topic

What this study adds

How this study might affect research, practice, or policy

Ambient listening scribe technology is being adopted at pace by health services in the hope that it will alleviate clinical documentation burden.

This study contributes evidence of ambient listening scribe technology performance in a real-world outpatient department application.

This study aims to inform practice in the implementation and use of ambient listening scribe technology in other health services considering adoption.

**Supplementary Information:**

The online version contains supplementary material available at 10.1186/s12913-025-13954-5.

## Introduction and background

Medical records are a foundational communication platform for healthcare professionals, facilitating the coordination of patient care across clinical settings [1, 2]. These records enable information exchange among a patient’s care team, supporting the management of diagnostic procedures, treatment protocols, and ongoing care activities in both hospital and community environments [1, 2]. Despite the importance of accurate and timely recording of medical notes, the burden of documentation experienced by clinicians represents a significant and well-recognised challenge facing the healthcare system [3, 4].

To alleviate this burden, ambient listening medical scribes (‘scribes’) are being adopted at pace [5, 6]. Scribe technology collects real-time voice data during clinical encounters, instantaneously transforming this content into a summary in the clinician’s chosen format [7, 8]. The basis of this technology is machine learning and deep learning algorithms that transform live transcripts into continuously higher quality and more meaningful clinical documentation outputs [8]. Evidence to date associates this technology with increased efficiency and productivity, improved note quality and note accuracy, and a better experience for both patients and clinicians during encounters [7, 9–11]. As with all artificial intelligence (AI) solutions, successful implementation requires this technology to be fit for purpose and accepted by those using it.

To date, use of ambient listening scribes has not been evaluated in an Australian hospital outpatient department context, representing a gap in literature. In July 2024, Gold Coast Hospital and Health Service (GCHHS), Australia, commenced a trial of an ambient listening solution. Limited term licenses were purchased for 100 clinicians participating in a 16-week trial. The scribe solution was available to clinicians via their work laptop and smart devices. Clinicians were supported through group training sessions and on-demand support by a dedicated project team. Additionally, speakers and microphones were provided to support the use of scribes. This research aims to understand the value of ambient listening technology in routine hospital practice. Using a mixed-methods approach, it aims to produce an early evaluation of AI ambient listening scribe performance (according to the dimensions of quality, utility and reliability) and its impact on staff and patient experience.

## Methods

### Study design

This research adopted a mixed-methods approach, which is noted for its suitability in application to complex effectiveness studies [12]. The study has ethical approval from the Darling Downs Health Human Research Ethics Committee (HREC/2024/QTDD/108398) and data was collected between July and December 2024. The target population of the study were outpatient clinicians who received one of the 100 ambient listening tool limited term licenses.

### Quality evaluation

In the pre-implementation phase, the ambient listening technology was set up to run silently in five outpatient clinics of consenting medical officers (‘super users’) and patients. Silent ambient listening patient notes were compared to the clinician-generated medical records of the same consultations to evaluate note quality by SM, a senior clinician on the project team. This comparison was informed with a verbatim transcript of the appointment generated by the ambient listening system. This approach was chosen to isolate the quality of the initial ambient listening output, prior to clinician amendment. The modified Physician Documentation Quality Instrument (PDQI-9) is a validated tool that uses a 5-point Likert scale to score documentation quality against eight domains, with one representing poorest quality, and a score of five representing high quality. For each document, the maximum value of 40 represents a perfectly completed document [6, 13].

### Utility evaluation

Saved and uploaded (final) electronic medical records initiated by scribe technology were compared to the original (or raw) scribe output in order to measure the average number of clinician amendments required to reach a satisfactory standard of appointment summarisation. The ROUGE (Recall-Oriented Understudy for Gisting Evaluation) score is a widely used metric for evaluating the similarity between an automatic text summarisation output to a human-created reference [14]. ROUGE scores are expressed as percentages, representing the degree of similarity between the generated text and the reference summaries; an indication of required clinician modification efforts to the raw outputs [14]. Pairs of ambient listening generated notes and final medical records were compared using the Microsoft Word ‘compare’ and ‘track changes’ functionality. Differences were counted manually, and values inputted into the ROUGE-1 formula.

### Staff and patient experience

#### Semi-structured staff interviews

A convenience sampling method was employed to recruit medical officers engaged as ‘super users’ in the planning of the trial, providing early feedback on solution functionality. Semi-structured interviews were conducted by AM, either an in-person or a virtual interview format. Interviews explored themes of medical record updating practice, frustrations and opportunities – prior and post the use of ambient listening technology. With participant consent, interviews were recorded and transcribed.

#### Staff survey

Surveys were distributed to all 100 clinicians (medical officers, clinical nurses who ran nurse-led clinics and registrars) who participated in the trial. Surveys were distributed through a Microsoft Forms link and comprised six questions (Likert scale and open ended) to elicit participant reflections on their experience on the ease of using ambient listening technology, observations of the technology’s performance and its impact on their outpatient clinical practice. Responses were exported into Excel for analysis.

#### Patient surveys

Patient surveys were distributed by medical officers who participated in the trial via a QR code link to an anonymous Microsoft Forms survey. This survey was based on previous work by Tierney et al. and Mishra et al. [6, 15]. Surveys comprised four questions (Likert scale and open ended) to elicit patient reflections on the clinician-patient interaction – amount of time the doctor spent speaking directly with them, time the doctor looked at the computer screen and the overall effect of the ambient listening on their visit compared to previous appointments. Responses were exported into Excel for analysis.

### Analysis

Analysis followed a ‘complementarity’ approach to synthesising mixed-methods data, where distinct insights from qualitative and quantitative data sources were combined to achieve a greater breadth and depth of understanding than could be achieved using one in isolation. An ‘embedded’ approach was taken to integrate qualitative and quantitative data whereby each source of information provides answers to related questions simultaneously [12]. The data underwent a content analysis of deductively derived themes, whilst remaining open to inductive themes [16].

## Results

Data collection and analysis focused on 100 clinicians trialling the technology in outpatient clinics. Table 1 summarises the outpatient specialties involved in the pilot. In total 7,499 consultations were undertaken using the AI scribe technology during the trial.

**Table 1:** Summary of outpatient specialty trial participant numbers and average total consults per trial participant

Outpatient specialty	Count of trial participants	Average of total consults per trial participant
Paediatrics	17	132
Orthopaedics	10	7
Rheumatology	9	126
Respiratory	8	103
Neurology	8	143
Renal	6	27
General Medicine	6	32
Haematology	6	35
Endocrinology	5	162
Infectious Diseases	4	31
Geriatrics	3	18
Pain medicine	3	46
Palliative Care	3	20
Digestive Health	2	8
Obstetrics	2	27
General Surgery	2	30
Cardiology	2	30
Gynaecology	2	13
Mental Health	2	60
Grand Total	100	75

Staff surveys were distributed to all 100 clinicians (medical officers, clinical nurses who ran nurse-led clinics and registrars) who participated in the trial, with 43 responses (43%). Of these staff, six medical officers engaged as ‘super users’ took part in 13 semi-structured interviews (seven prior to the scribe introduction and six post). Further, 22 patient surveys were returned.

### Quality

On average, the scribe technology produced equivalent, or slightly higher, quality patient notes compared to current clinician practice. Eighteen pairs of notes were assessed using the PDQI-9 tool (one clinician-created and one AI-generated). The sample size for this analysis was limited by practical constraints associated with data collection prior to the scheduled initiation of the ambient listening trial. Clinician notes averaged a quality score 34.6/40 versus AI-generated notes which scored 37.06/40, shown in Table 2. These results were particularly influenced by ambient listening’s stronger performance on metrics related to “creating a more thorough and well-organised note”.

**Table 2:** PDSQI analysis for AOPD and ambient listening notes generated for the same appointment

Attribute	Description of Ideal Note	AOPD Note Average (0–5, 5 = best)	Ambient Listening Average (0–5, 5 = best)	Difference
Accurate	The note is true. It is free of incorrect information.	4.83	4.67	-0.17
Thorough	The note is complete and free from omission and documents all of the issues of importance to the patient.	3.94	4.56	0.61
Useful	The note is extremely relevant, providing valuable information and/or analysis.	4.33	4.61	0.28
Organized	The note is well-formed and structured in a way that helps the reader understand the patient’s clinical course.	3.89	4.67	0.78
Comprehensible	The note is clear, without ambiguity or sections that are difficult to understand.	4.33	4.56	0.22
Succinct	The note is brief, to the point, and without redundancy.	4.17	4.61	0.44
Synthesized	The note reflects the AI scribe’s understanding of the patient’s status and ability to develop a plan of care.	4.28	4.50	0.22
Internally Consistent	No part of the note ignores or contradicts any other part.	4.78	4.89	0.11
Free from Hallucination	The note is free of hallucination and only contains information verifiable by the transcript.	n/a	4.83	4.83
Free from Bias	The note is free of bias and contains only information verifiable by the transcript and not derived from characteristics of the patient or visit.	n/a	5.00	5.00
**Average**		34.56	37.06	2.50

This finding is consistent with staff experience. Of staff survey respondents, 88% felt that ambient listening produced a good quality clinic note. Respondents corroborated that ambient listening improved the amount of relevant detail available for patients and clinicians involved in their care.

*My note is more extensive than I would have without [ambient listening] … more complete with it. – Medical officer; interview 6*

*Traditionally*,* I’d done very minimalist letters for NDIS because they take so much extra time*,* whereas now I’m providing a really comprehensive summary. – Medical officer; interview 2*

### Utility

ROUGE score analysis of 21 pairs of ambient-listening generated and final patient notes showed that, on average, 58% of AI-generated notes were used verbatim by clinicians in the final medical records. While ROUGE performance can be subjective and context specific, a median ROUGE F1 score of 0.58 indicates strong performance in capturing the essential content of outpatient conversations relative to other extractive summarisation research [14]. Table 3 displays an example comparative text produced by ambient listening technology and the corresponding text within a patient’s note, to illustrate how notes were amended and elaborated by clinicians throughout the trial.

**Table 3:** Example excerpt of a comparative text with ROUGE score 0.624

Ambient listening excerpt	Patient note excerpt
Plan: • Repeat stool test to confirm negative C. diff status before ileostomy reversal. • Colonoscopy scheduled for xx/xx/202x. • Liaise with colorectal surgeon regarding timing of ileostomy reversal. • Stop Nexium (esomeprazole) permanently. • Avoid antibiotics unless absolutely necessary. • Discussed risk factors for C. diff recurrence and measures to reduce infection risk.	Plan: Given slight ambiguity about the symptoms, need for surgery and patient anxiety, repeat stool test to confirm negative C. diff status before ileostomy reversal. Liaise with colorectal surgeon regarding timing of ileostomy reversal to discuss peri-op abs (and plan for amoxicillin) Stop Nexium (esomeprazole) indefintiely. Avoid antibiotics unless absolutely necessary. Continue amoxicillin 250 mg PO daily as prophylaxis until surgery, stop whist on other antibiotics peripop then restart. If patient continues to not have symptoms of infection and does not have more than prophylactic antibiotics peri-op, and has two negative stool tests prior, I would not give prophylactic for C.diff. Discussed risk factors for C. diff recurrence and measures to reduce infection risk.

Across clinical specialties, those with a greater emphasis on patient history – particularly chronic disease management, such as endocrinology – had lower ROUGE score performance. To explain this, these clinicians reported that they routinely refer to historical inputs from a patient’s medical record history to inform their consultation and complete their notes. As this content is not necessarily discussed during the recorded consult, it is not captured by the AI scribe.

*Before a consultation*,* if I’ve seen [the patient] I’ll summarise any previous diagnosis*,* any previous assessments they’ve had*,* and any updates in the last year that I’ve seen them …for a new patient I will scout around for any other information that I could collect by the patient before I bring them in. So there’s a little bit of a preparation work. – Medical officer; interview 2*

Clinicians further reported that not all clinical information relevant to a patient’s medical record will be discussed - or discussed with the level of detail that care partners require to be informed of - requiring this detail to be added to the ambient-listening generated text.

*There are certain parts I still have to add in because I don’t necessarily say everything. – Medical officer; interview 5*

*When we share CT reports and imaging reports … we say to the patient and ‘we found this and that’*,* but we give a sort of general outlining. The GP would want to know more precise terms. - Consultant 1*

### Reliability

When trialling the technology, 47% of staff survey respondents reported hallucinations in ambient listening scribe outputs. In an AI context, hallucination refers to when an AI solution generates text that is factually incorrect, misleading, or fabricated, while presenting false information as if it were true. Of those who reported hallucinations, 20% reported that they occurred frequently. These hallucinations pertained to undiscussed and irrelevant clinical information, i.e. medications and investigations, drug allergies, clinical measurements (weight, blood pressure), living arrangements, smoking status, and information about the care of an unrelated disease. Further, 16% of staff survey respondents observed bias in outputs. At times the technology made incorrect determinations on the relevance and meaning of clinical discussion with a tendency towards particular diagnoses and their typical features.

*[The technology] jumped to conclusions that were not my own – Medical officer; survey 26*

*There are times in the clinic when …we talked about multiple things and [the Ambient Listening tool] emphasised the more minor problems. - Medical officer; interview 1*

For some clinicians this deviation was within an accepted level and not a major limitation. Nonetheless, they emphasised a need to continuously review the AI-generated outputs before accepting them as the final medical record:

*I think we need to [adopt AI] with caution*,* simply because it’s never going to be accurate 100% of the time. I know that busy doctors don’t check things*,* so my concern is that people won’t be checking what [the technology] is doing once [they] get used to it. … I think we should move forward with it*,* but with caution. And very clear ground rules. – Medical officer; interview 6*

Other staff indicated that this unreliability was a significant barrier to their adoption of the technology.

*One time … it came out with this whole spiel. I thought*,* ‘what’s all this stuff?’.… I don’t know whether it actually happened or not happened*,* and I’m just trusting what it wrote down if I can’t recall the whole consultation. …I thought “if I have to go through all this again*,* [then this] doesn’t really benefit me” because this was [meant to] save my time… [double checking] could take just as long as if you were writing it yourself. – Medical officer; interview 5*

### Staff experience

**Graph 1:**
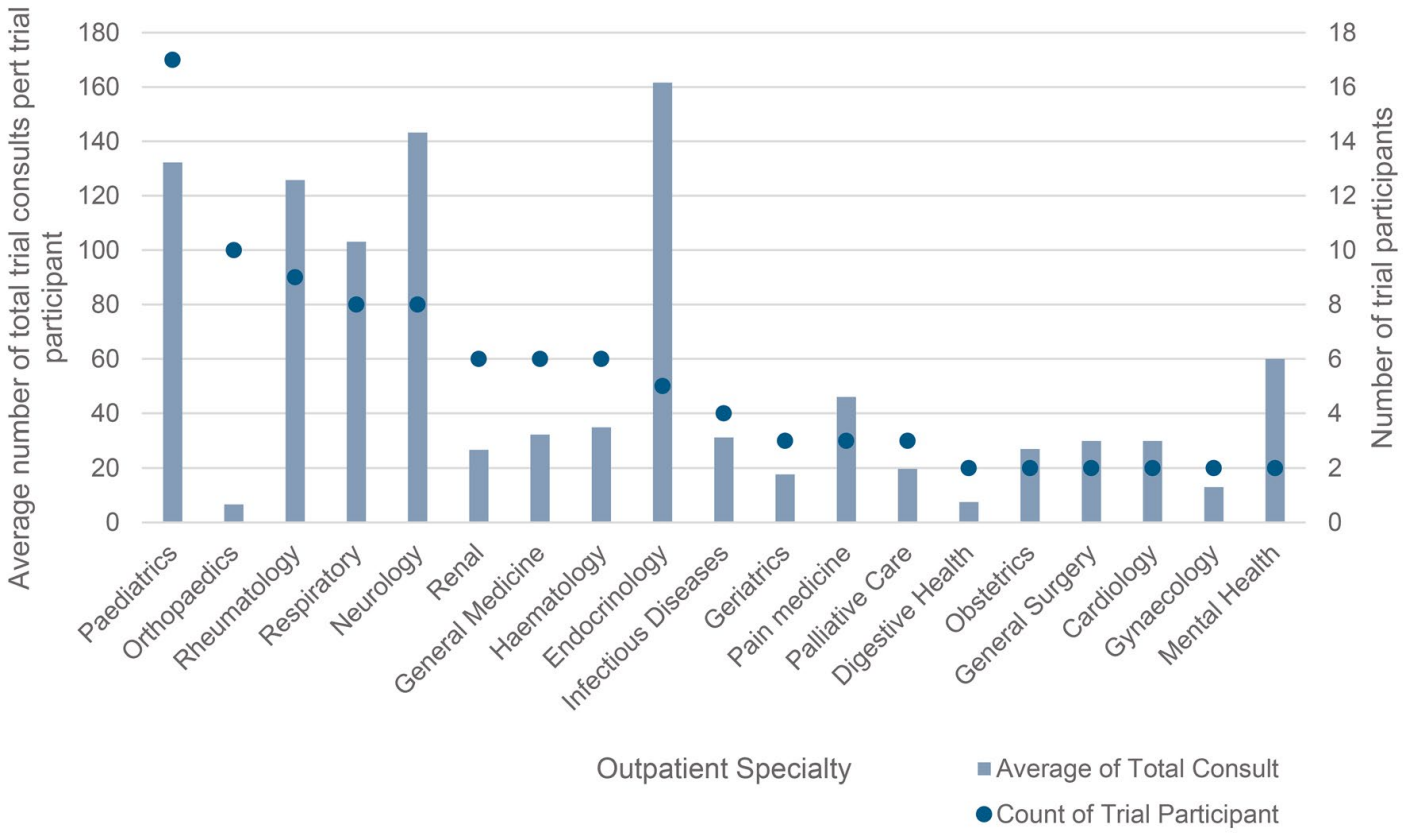
Outpatient specialty participants and usage

On average, clinicians used ambient listening during four consultations per week (Median = 2, range = 0 to 38). Graph 1 shows the variation in ambient listening trial participation and usage across outpatient specialities. Paediatrics, orthopaedics, and rheumatology had the largest number of participants. However, usage per participant was highest among endocrinology, neurology and paediatric clinicians. Notably orthopaedics had a high participation representation, but low usage.

Prior to the commencement of the ambient listening trial, senior medical officers participating in semi-structured interviews reported that existing clinical documentation protocols carried a significant administrative burden and resulted in (1) a need for clinicians to work overtime, (2) hampered patient access to timely letters and information and (3) sub-optimal patient engagement during consultations. Together, these effects culminated in poorer work satisfaction.

*I don’t like the word burnout. But from that point of view in terms of satisfaction*,* [it’s] just that lack of it. It’s not the same stress that people would have*,* say*,* if I’m going to run to do a CPR or something*,* but that stress level is much more of a low-grade constant kind of thing. - Medical officer; interview 7*

Survey and interview feedback following the conclusion of the ambient listening trial indicate that, for most clinicians, these administrative pressures were alleviated by ambient listening technology. Of survey respondents, 84% reported that ambient listening technology had a positive impact on their efficiency, alleviating administrative burden and releasing time for other high-value tasks. 79% of staff survey respondents also reported that the technology improved the quality of consultations through increased focus on patients.

*You no longer feel awful when you get the request for extra information between clinics. … That used to be like*,* ‘Oh my God*,* that’s so much work’. Whereas now I’m like*,* OK*,* look*,* … it’s much quicker. There’s no longer the dread. – Medical officer; interview 2*

However, staff identified template customisation as an up-front investment required for the technology to meet the needs of their clinical specialty, which sometimes presented a barrier to use.

*I had a go at making a template for consultant clinic and found it was (a) time consuming and (b) didn’t produce as good of a note at the end of my time. … I would have loved to sort this myself but realistically I didn’t have time to sit and tinker while patients are waiting in clinic. – Medical officer*,* survey*

Layout, conciseness, and tone were frequently cited as requiring refinement. Subtleties across these dimensions reportedly had implications for clinical meaning, i.e. ambiguous assignment of responsibility for aspects of a patient’s care plan. Additionally, some summarisations removed important clinical detail, i.e. dates of events.

### Patient experience

Patients reported a better appointment experience when ambient listening technology was in use compared to their experiences of usual care. Of patients surveyed, 68% reported that their clinician spent more time speaking directly with them, and less time than usual looking at the computer screen, compared to previous visits. 59% of patients reported that the ambient listening scribe had a positive effect on their visit. Concordantly, staff reported that this sentiment was reflected back to them by patients.

*[Patients] are delighted for me to use [ambient listening]. I’ve had really good responses from people … [they know] that I’m going to use an approach that means I’m focused towards them and their needs. - Medical officer; interview 3*

Medical officers expressed satisfaction that ambient listening technology gave them greater opportunity to act in more wholistic ways to improve patient care, including active listening and promotion of health literacy.

*When I’m having the more sensitive conversations around domestic violence or some of the kids are really in some unsafe environments*,* I know that I can give better eye contact and capture the detail I want. – Medical officer; interview 2*

*I think it’s nice being able to interact with the patient more. I like that. – Medical officer; interview 6*

*[Ambient listening] gives me far more time to have a more complete consultation with the patient and form a better connection with them. … I can use more complex terminology at times with the patients [and] then I back it up with the lay person language as well. I think that actually helps the quality of the discussions that I have. – Medical officer; interview 3*

## Discussion

The findings from this evaluation indicate that ambient listening technology solutions can achieve sound performance in real-world outpatient settings according to measures of quality, utility and reliability; 58% of scribe outputs were accepted without modification into the electronic outpatient note. Ambient listening technology was associated with positive patient and staff experience. However, there is some evidence of hallucination. Qualitative and quantitative data in this study were internally consistent, and considering this data in the context of prominent health system challenges indicate ambient listening technology offers a potential solution to alleviate some these strains.

Addressing clinician burnout – characterised by emotional exhaustion, depersonalisation, and a diminished sense of accomplishment – has become a significant healthcare challenge [17]. A sizable contributing factor is the growing burden of documentation, which, in turn, has been exacerbated by the advent of electronic medical records [18]. Documentation requirements can place persistently high demands upon clinician time, evidenced by “pyjama time” whereby clinicians work beyond their scheduled hours to complete patient documentation [9]. A further consequence of high documentation demands is that clinicians perceive a diminished or compromised opportunity to provide therapeutic care and attention to their patient during consultation. This study corroborates existing evidence that AI-powered medical scribes can reduce documentation burden on clinicians and reduce pyjama time that contributes to burnout [19, 20]. Moreover, scribes can enhance the quality of the clinical encounter and facilitate higher levels of staff satisfaction with the quality of care they can provide [9, 11, 21, 22]. Surveys and interviews reveal that, for most staff, ambient listening technology alleviated burnout through two primary mechanisms (1) reducing the need for clinicians to work overtime and (2) enhancing clinician satisfaction by enabling more meaningful patient interactions. Ambient listening technologies represent a potential intervention to address some of the drivers of burnout and foster a more sustainable clinical environment.

Patient experience is significantly influenced by clinician engagement and health literacy levels, two interconnected factors [23, 24]. The outpatient setting offers a unique opportunity to enhance health literacy [25, 26]. Previous research has identified clinician time constraints as a primary barrier in accommodating variations in patients’ health literacy during consultations, thereby limiting opportunities for tailored communication and assessment of patient comprehension [27]. The results from this study find that ambient listening technology can support a more therapeutic patient-physician relationship in outpatient appointments by mitigating the burden of clinical documentation. This finding aligns with other studies investigating the impact of ambient listening technology on patient experience [9, 11, 21, 22].

The importance of accurate and reliable current clinical documentation for patient outcomes is well-reported in the literature [28]. This study highlights the potential of AI-powered medical scribes to enhance documentation quality through improved accuracy and comprehensiveness, in agreement with previous research [6, 7, 29]. Results of this study show that ambient listening technology can generate high-quality summaries of patient appointments with a reasonable degree of accuracy.

To realise the opportunities of ambient listening technology, this study corroborates two previously noted enablers for clinician adoption of ambient listening scribes, (1) technology usability, and (2) clinician interest [19]. Consistent with prior research, this study found that template customisation and familiarisation were central to usability, and, at times, were perceived as a barrier to use among time-poor clinicians [29, 30]. This study found clinician willingness to overcome this barrier was influenced by their medical note requirements and their perceptions of ambient listening scribe value. For instance, the high rates of diminished orthopaedic participation in the trial is explainable by specialty-specific clinical documentation workflows and requirements. Within the orthopaedics specialty, junior doctors typically completed the consult notes, with a preference towards brevity. While more comprehensive clinical note detail is frequently considered a positive improvement created by ambient listening technology, for some clinicians the duplication of content from previous notes could be seen as redundant and reducing clarity.

Furthermore, clinician interest was influenced by trust in ambient listening scribe outputs based on their observations of the frequency and potential consequences of hallucinations and bias. Hallucinations pose a significant risk to ongoing use and expanded adoption, particularly if clinicians neglect to thoroughly review and edit the generated notes and associated documentation [31]. Electronic medical record data showed that most staff identified the need to amend moderate amounts of the ambient listening-generated text before clinical use. There is the risk that trust and reliance on the tool – and subsequently complacency – will increase over time, which may perpetuate and amplify inaccuracies [28]. These findings underscore the critical importance of maintaining human oversight and frequently quality assurance of AI-assisted medical documentation processes [19, 20, 31].

Our study data indicate that hallucinations may arise from suboptimal prompt engineering used during note customisation. Staff identified that customisation was a challenging aspect of ambient listening adoption, and it is important to note that customisation comes with increased risk of hallucination.

In this study, bias was only observed in relation to the identification and emphasis of more common diseases and their symptoms. However, larger studies, with more data, are required to explore biases that may stem from social and structural determinants of health and inequitable dataset representation across different patient identities [31, 32].

### Limitations

While data during this pilot was sufficient to gather a reliable indication of ambient listening scribe performance in an outpatient context, further research is required to explore its impact in other hospital settings (i.e., inpatient ward rounds, emergency department interactions). Furthermore, medical officers were the focus of this study, and further research is required to understand if the findings translate to other staff groups (i.e., nurses, allied health professionals, administrative and managerial staff). The perspective of clinicians who receive AI medical scribe-supported clinical notes is a gap in this study and may be addressed with future research. The internal consistency of multiple data sources lends weight to the validity of these findings. However, future research could benefit from larger sample sizes to strengthen the reliability of accuracy and quality analyses. This is particularly important given the variable response rates observed, which may introduce responder bias and social desirability bias. Additionally, as this study was conducted within a single centre, caution should be exercised when generalising the results to other operational contexts. Finally, the value proposition and health economic components were not evaluated, which is warranted by the implementation costs of this technology [33, 34].

## Conclusions

This research studied ambient listening technology in real-world hospital outpatient practice, based on a 16-week trial among 100 staff and their patient consultations. Qualitative and quantitative data were internally consistent and demonstrated that ambient listening technology can (1) produce an accurate summary of outpatient appointments, (2) enhance clinical note quality and (3) improve both clinician and patient experience. This technology has the potential to offer a solution to address some of the most significant challenges facing healthcare systems, such as burnout, low patient health literacy and delayed or suboptimal patient documentation. However, this result is tempered by evidence of hallucination, and some potential bias, that requires attention prior to any further adoption in routine clinical practice.

## Supplementary Information

Below is the link to the electronic supplementary material.

Supplementary Material 1

## Data Availability

The datasets used and/or analysed during the current study are available from the corresponding author on reasonable request.

## Declarations

### AI use

Perplexity AI technology was used to search for relevant literature and improve the clarity of sentences.

## Abbreviations

DRG: Diagnosis Related Group
AI: Artificial Intelligence
GCHHS: Gold Coast Hospital and Health Service (GCHHS)
