# Supplementary material for: Performance, acceptability, and impact of ambient listening scribe technology in an outpatient context: a mixed methods trial evaluation
Source: BMC Health Serv Res. 2026 Jan 8;26:182. doi: 10.1186/s12913-025-13954-5 (PMC12882620; doi:10.1186/s12913-025-13954-5)
Supplement: Supplementary file 1 — Supplementary Material 1 [file 12913_2025_13954_MOESM1_ESM.pdf]

Supplementary File 1_ Ambient Listening Questionnaires Combined

Survey Questions – Patients

An evaluation of Ambient listening technology - Patient survey

Introduction: Thank you for your time to provide your feedback on your experience at today's outpatient appointment. This short survey is anonymous and voluntary. Its purpose is to see if a new piece of technology that was in use during your appointment (ambient listening) improved your experience when discussing your care with your clinician. This survey is expected to take no longer than 1 minute.

Compared to typical previous visits with your doctor, in this visit that included a scribe in the room:

1. Did you notice any difference in the time your doctor spent speaking directly with you?

Time spent speaking directly with you:		
Less time than usual<br>☐	No difference<br>☐	More time than usual<br>☐

2. Did you notice any difference in the time your doctor spent looking at the computer screen?

Time looking at the computer screen:		
Less time than usual<br>☐	No difference<br>☐	More time than usual<br>☐

3. In your view, what effect (if any) did the scribe have on your visit?

Quality of your visit:				
Much worse<br>☐	Somewhat worse<br>☐	No effect<br>☐	Somewhat better<br>☐	Much better<br>☐

Survey Questions – Staff

An evaluation of Ambient listening technology - Clinician survey

Q.1. On a scale of 'strongly agree' to 'strongly disagree' (or n/a) [strongly agree, agree, slightly agree, neutral, slightly disagree, disagree, strongly disagree, N/A], please rate your agreement with the following statements:

Q.1.1 Ambient listening is intuitive and easy to use.

Q.1.2 If available, I would continue to use ambient listening after this trial.

Q.1.3 Ambient listening improved timely completion of outpatient department tasks, i.e. reducing post-outpatient notes and letter finalisation.

Q.1.4 Ambient listening improved the quality of consultations by allowing me to spend more time talking to the patient as opposed to focusing on completing clinical notes.

Q.1.5 It is easy to cut/copy & paste information from ambient listening into ieMR, SWT and CIMHA.

Q.1.6 It is easy to amend ambient listening drafted correspondence after the consultation.

Q.1.7 It is easy to sign into the app via the GCH network.

Q.1.8 Ambient listening can listen to multiple attendees at the same time and distinguish between clinician and patient (and relevant supporting attendees).

Q.1.9 Ambient listening only includes relevant clinical information and removes non-medical chatter from the transcription.

Q.1.10 Ambient listening uses punctuation correctly.

Q.1.11 Ambient listening produces a good quality letter to GP.

Q.1.12 Ambient listening's patient consent workflow is easy to use.

Q.1.13 Ambient listening accurately records acronyms (e.g., GCS = Glasgow coma scale).

Q.1.14 Ambient listening accurately records speech with regional and foreign accents.

Q.1.15 Ambient listening performs well when using devices other than a mobile phone i.e., WOW, Webcam.

Q.1.16 Ambient listening performs well when using Wi-Fi.

Q.1.17 Ambient listening integrates well with the existing letters to GP workflow in iEMR.

Q.1.18 Ambient listening support and training has been sufficient.

Q.2. 'Hallucinations' in ambient listening is false information generated without sound basis. During your time in the ambient listening trial, have you observed any hallucination in ambient listening transcripts?

Q.2.a. If so, please describe the instances ___.

Q.3. 'Bias' in ambient listening is information generated based on use of discriminatory data, algorithms, or faulty heuristics. During your time in the ambient listening trial, have you observed any bias in ambient listening transcripts?

Q.3.a. If so, please describe the instances ___.

Q.4. Do you feel that the tool has improved/increased efficiency?

If yes, how?

If no, what would need to be improved to increase efficiency?

Q.5. On a scale of simple, moderate, or extensive, please rate the quality of transcription based on the number of "edits" that you had to make on ambient listening-generated EMR notes.

Q.6. Do you have any other comments, or feedback as to how the tool could be improved?

Interview Questions – Staff

Pre-phase Ambient listening Evaluation Clinician Semi-Structured Interview Questions

1. What is your current process to update an outpatient's electronic medical record?
2. What aspects of the current process are working well?
3. What aspects of the current process cause you furstration?
4. On average, how long does it take to update an electronic medical record for one patient following an outpatient appointment?
5. In an ideal world, following current processess, how much time would you have available to you to update an electronic medical record for one outpatient appointment?
6. What barriers do you currently experience to update patient electronic medical records to your ideal standard?
7. What is the impact of these barriers on timely completion of outpatient department tasks, such as post-OPD note/letter finalisation?
8. Are there any other impact of these barriers? ?
    - Prompt: on the quality of your updates?
    - Prompt: on turnaround time for letters to go to general practitioners?
    - Prompt: on timely completion of outpatient department tasks, such as post-OPD note/letter finalisation?
    - Prompt: on patient care continuation?
    - Prompt: on your level of stress?
    - Prompt: on your ability to manage your workload?
    - Prompt: on your ability to complete other important tasks (such as business process improvement activities)?
9. What are your thoughts on applying AI to support your electronic medical record updates?
    - Do you have any concerns?

Post-phase Ambient listening Evaluation Clinician Survey

1. How do you use Ambient listening to update an outpatient's electronic medical record?
    - Do you review notes taken by Ambient listening?
    - Do you need to adjust the template?
    - Do you need to adjust the language?
2. What aspects of the Ambient listening process are working well?
3. What aspects of the Ambient listening process cause you furstration?
4. On average, how long does it take to update an electronic medical record for one patient following an outpatient appointment using Ambient listening?
5. In an ideal world, using Ambient listening, how much time would you have available to you to update an electronic medical record for one outpatient appointment?
6. Has Ambient listening addressed any of the barriers you previously cited to update patient electronic medical records to your ideal standard?
    - If so, which ones?
7. What has been the impact of addressing these barriers on timely completion of outpatient department tasks, such as post-OPD note/letter finalisation?
8. Have there been any other impact of addressing these barriers?
    - Prompt: on the quality of your updates?
    - Prompt: on patient care continuation?
    - Prompt: on your level of stress?

- Prompt: on your ability to manage your workload?

9. What barriers do you (still) currently experience to update patient electronic medical records to your ideal standard?
10. What is the impact of these barriers?
    - Prompt: on the quality of your updates?
    - Prompt: on patient care continuation?
    - Prompt: on your level of stress?
    - Prompt: on your ability to manage your workload?
    - Prompt: on your ability to complete other important tasks (such as business process improvementa activities)?
11. What are your thoughts now on applying AI to support your electronic medical record updates?
    - Do you have any concerns?
12. Since you have begun using Ambient listening, have you observed a change in the quality of your clinical handover notes and/or letters?
13. Have you used or observed the use of the data generated by Ambient listening to drive any new models of care and workflows?
14. What, if any, has been the impact of Ambient listening on your relationships with patients' ongoing care providers
    - Prompt: on referrals and care continuation?
15. Have you observed any other value from having access to real-time EMR data produced by Ambient listening?
